# Supplementary material for: Whole genome sequencing and single-cell transcriptomics identify KMT2D inactivation as a potential new driver for pituitary tumors: a case report
Source: BJC Rep. 2025 Jun 16;3:43. doi: 10.1038/s44276-025-00155-0 (PMC12170844; doi:10.1038/s44276-025-00155-0)
Supplement: Supplementary file 1 — suppmat.Case_report_KMT2D [file 44276_2025_155_MOESM1_ESM.docx]

Supplementary Material:

Whole Genome Sequencing and single-cell transcriptomics identify *KMT2D* as a potential new driver for pituitary tumors: a case report.

Maxime Brunner^1,2^, Jeny Meylan-Merlini^1^, Maude Muriset^1^, Sergey Oreshkov^1,2^,

Andrea Messina^1,2^, Mahmoud Messerer^2,3^, Roy Daniel^2,3^, Ekkehard Hewer^2,4^, Jean

Phillipe Brouland^2,4^, Federico Santoni^1,2,*^

1. Department of Endocrinology, Diabetology and Metabolism, University Hospital of Lausanne, Switzerland
2. Faculty of Biology and Medicine, University of Lausanne, Switzerland
3. Department of Neurosurgery, University Hospital of Lausanne, Switzerland
4. University institute of Pathology, University Hospital of Lausanne, Switzerland

*Correspondence to: federico.santoni@chuv.ch

| Patient | Tumor type | Sex | scRNA | WGS | Methylation | Figure |
| --- | --- | --- | --- | --- | --- | --- |
| Patient 1 | GH1-PRL | Male | x |  |  | 4 |
| Patient 2 | POMC | Female | x |  |  | 4 |
| Patient 4 | NS | Male | x |  |  | 4 |
| Patient 7 | NS | Male | x | x |  | 3,4 |
| Patient 9 | NS | Male | x |  |  | 4 |
| Patient 10 | NS | Male | x | x |  | 3,4 |
| Patient 11 | POMC | Female | x | x |  | 3,4 |
| Patient 12 | GH1-PRL | Female | x | x |  | 3,4 |
| Patient 13 | PRL | Female | x | x | x | 3,4,5 |
| Patient 16 | GH1-PRL | Male | x | x | x | 1,2,3,4,5 |
| Patient 18 | POMC relapse | Female | x | x | x | 5 |
| Patient 20 | NS relapse | Male |  | x | x | 5 |
| Patient 21 | POMC | Female |  | x | x | 5 |
| Patient 22 | GH1-PRL + gangliocytoma | Female | x | x | x | 4,5 |
| Patient 25 | PRL | Female |  | x | x | 5 |
| Patient 26 | NS | Female |  | x | x | 5 |
| Patient 28 | GH1 | Female |  | x | x | 5 |
| Patient 29 | GH1 | Male |  | x | x | 5 |
| Patient 30 | unknown | Female |  | x | x | 5 |
| Patient 31 | NS | Male |  | x | x | 5 |
| Patient 37 | unknown | Female |  | x | x | 5 |
| Patient 40 | POMC | Female |  | x | x | 5 |
| Patient 42 | GH1 | Female |  | x | x | 5 |
| Patient 44 | NS | Female |  | x | x | 5 |

Supplementary Table 1: All PitNETs used in this study. GH1-PRL: mixed somato-lactotroph tumor, POMC: corticotroph tumor, NS: non-secreting tumor, PRL: lactotroph tumor, SC: singlecell, WGS: whole genome sequencing.


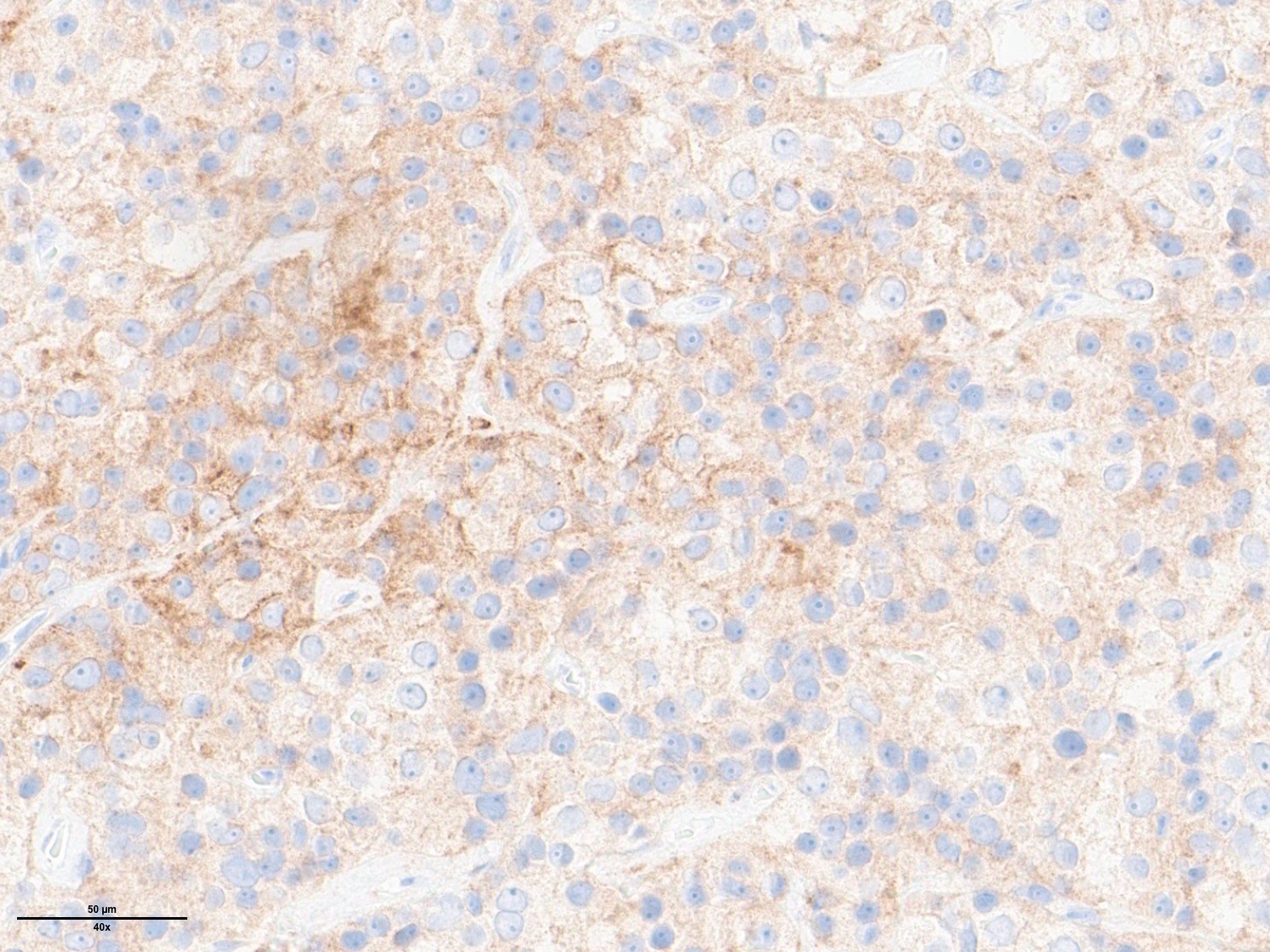

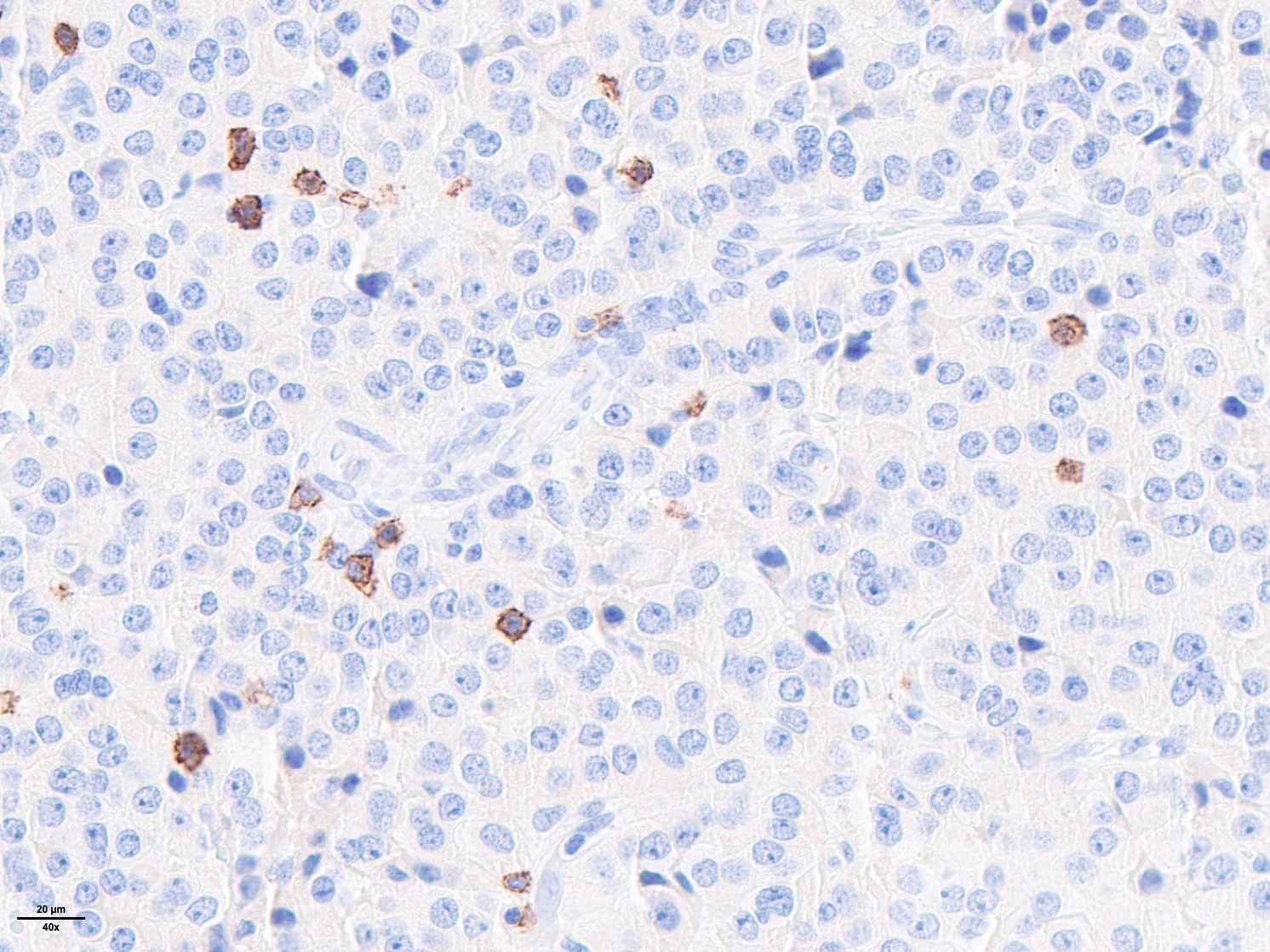

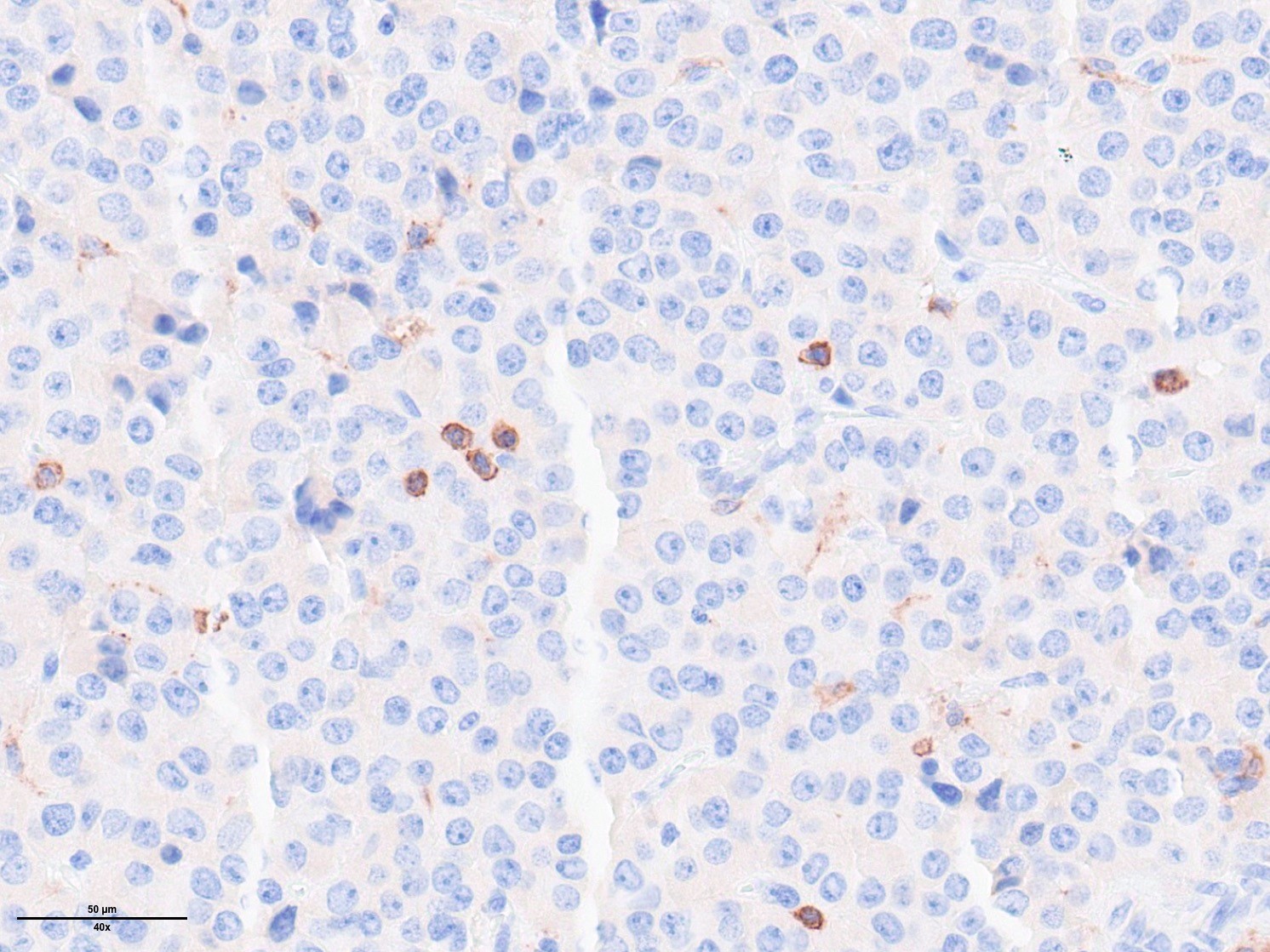

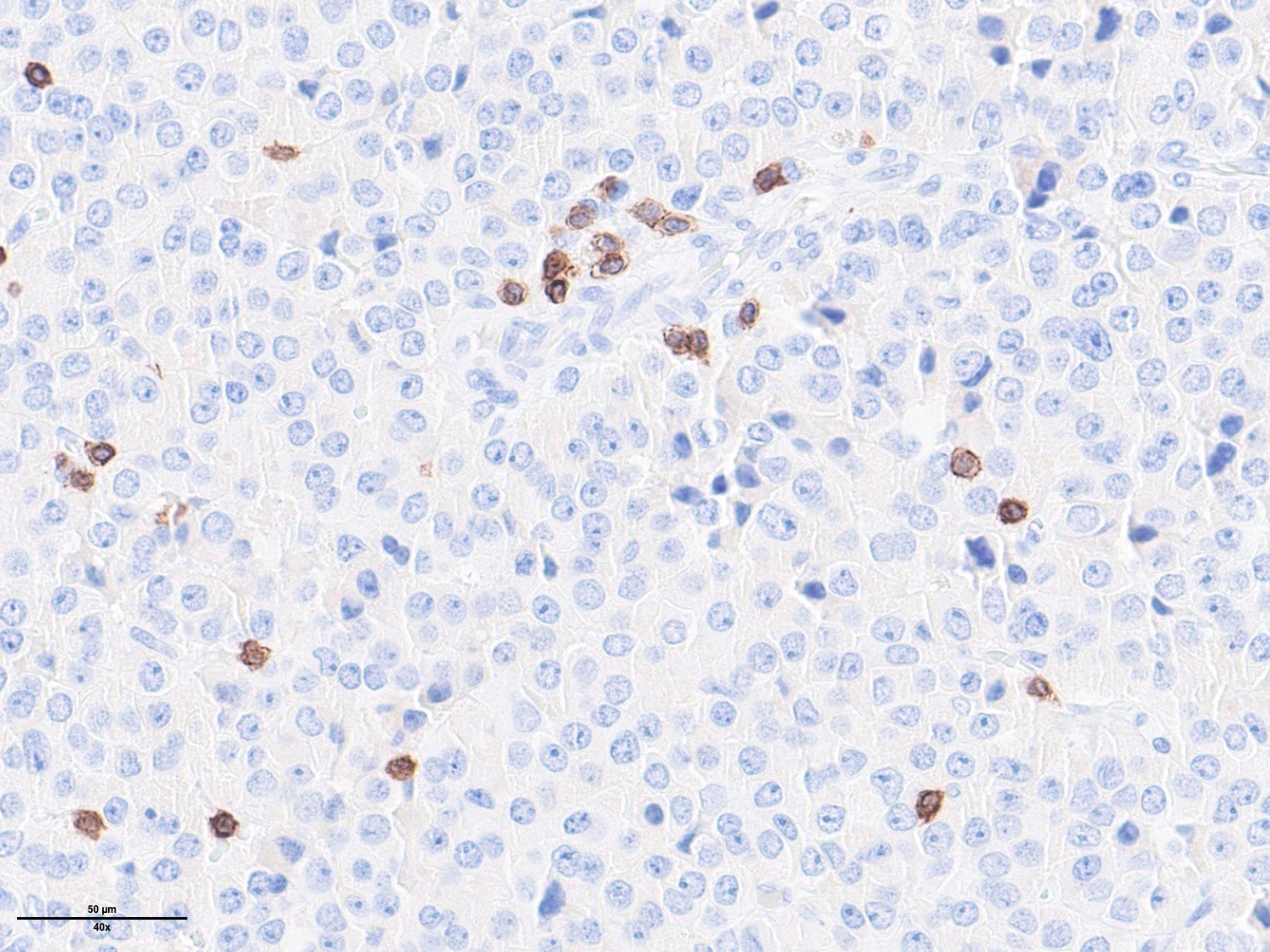


**A**

**B**

**D**

**C**

**CD3**

**CD8**

**CD4**

**PD-L1**


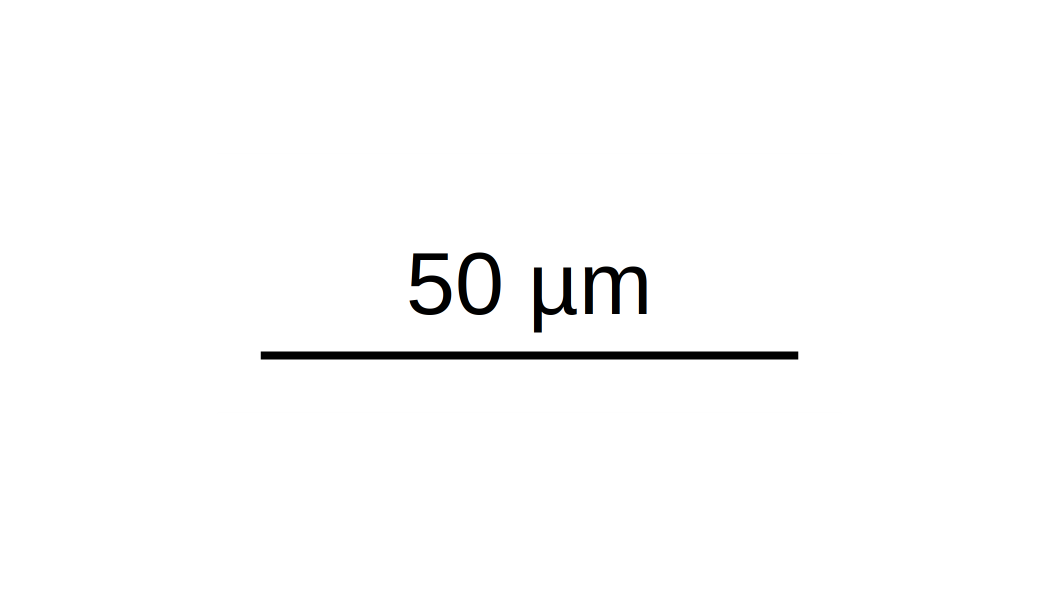

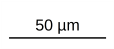

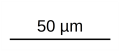

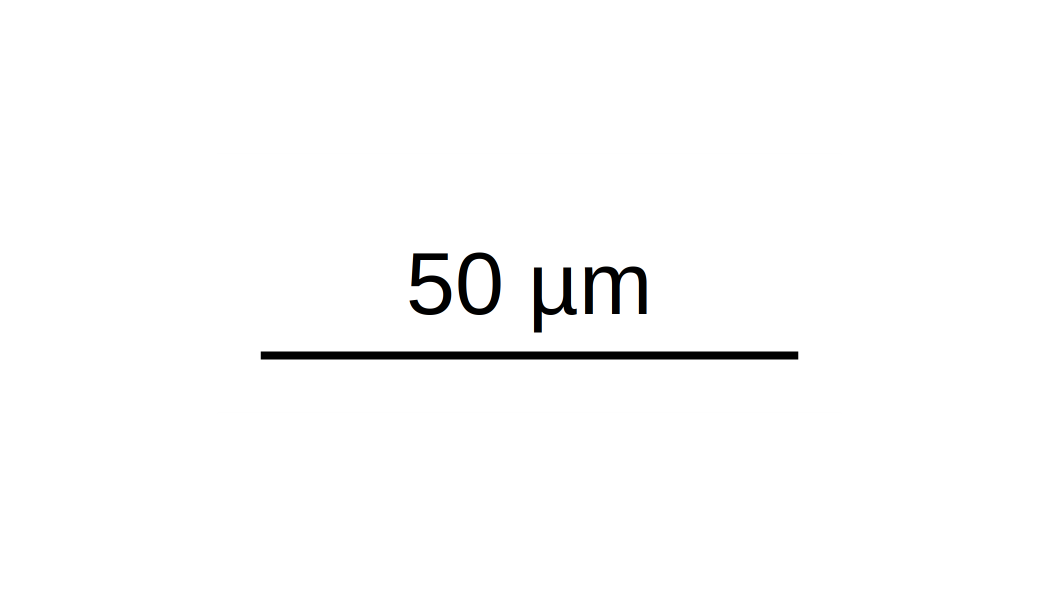

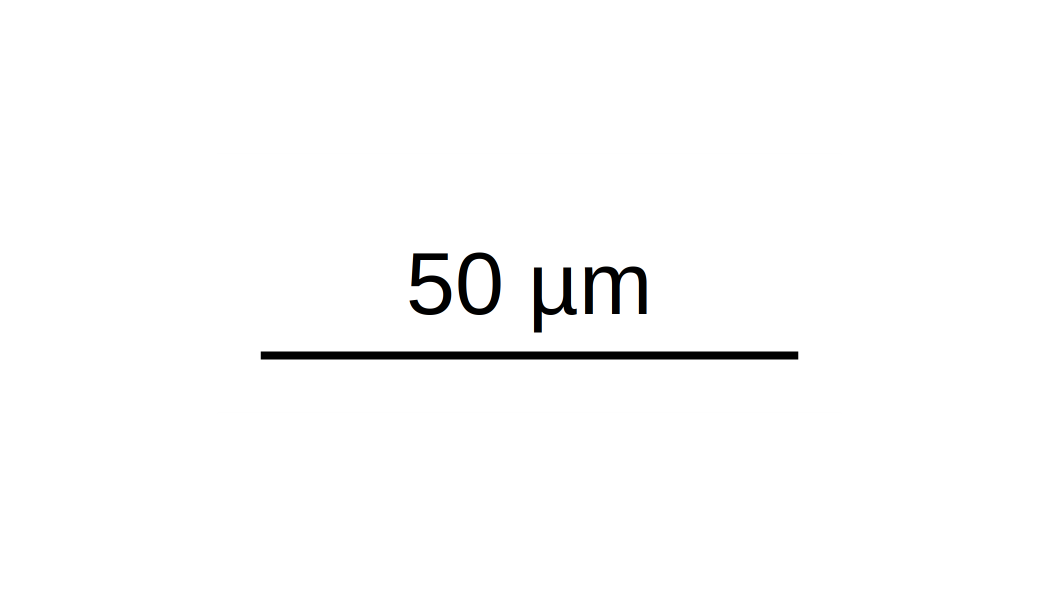


Supplementary Figure 1: **A-C**: Immune cell markers staining. **D**: Check point inhibitor PD-L1 staining
